# Characterization of *Staphylococcus aureus* Cas9: a smaller Cas9 for all-in-one adeno-associated virus delivery and paired nickase applications

**DOI:** 10.1186/s13059-015-0817-8

**Published:** 2015-11-24

**Authors:** Ari E. Friedland, Reshica Baral, Pankhuri Singhal, Katherine Loveluck, Shen Shen, Minerva Sanchez, Eugenio Marco, Gregory M. Gotta, Morgan L. Maeder, Edward M. Kennedy, Anand V. R. Kornepati, Alexander Sousa, McKensie A. Collins, Hari Jayaram, Bryan R. Cullen, David Bumcrot

**Affiliations:** Editas Medicine, Cambridge, MA 02142 USA; Department of Molecular Genetics and Microbiology and Center for Virology, Duke University Medical Center, Durham, North Carolina 27710 USA

**Keywords:** CRISPR, Cas9, Genome engineering, Nickases, *Staphylococcus aureus*, Adeno-associated virus, GUIDE-seq

## Abstract

**Background:**

CRISPR-Cas systems have been broadly embraced as effective tools for genome engineering applications, with most studies to date utilizing the *Streptococcus pyogenes* Cas9. Here we characterize and manipulate the smaller, 1053 amino acid nuclease *Staphylococcus aureus* Cas9.

**Results:**

We find that the *S. aureus* Cas9 recognizes an NNGRRT protospacer adjacent motif (PAM) and cleaves target DNA at high efficiency with a variety of guide RNA (gRNA) spacer lengths. When directed against genomic targets with mutually permissive NGGRRT PAMs, the *S. pyogenes* Cas9 and *S. aureus* Cas9 yield indels at comparable rates. We additionally show D10A and N580A paired nickase activity with *S. aureus* Cas9, and we further package it with two gRNAs in a single functional adeno-associated virus (AAV) vector. Finally, we assess comparative *S. pyogenes* and *S. aureus* Cas9 specificity using GUIDE-seq.

**Conclusion:**

Our results reveal an *S. aureus* Cas9 that is effective for a variety of genome engineering purposes, including paired nickase approaches and all-in-one delivery of Cas9 and multiple gRNA expression cassettes with AAV vectors.

**Electronic supplementary material:**

The online version of this article (doi:10.1186/s13059-015-0817-8) contains supplementary material, which is available to authorized users.

## Background

The initial characterizations of CRISPR-Cas systems as mechanisms of bacterial and archaeal adaptive immunity [1–3] led to demonstrations of guide RNA (gRNA)-targeted DNA cleavage in vitro by the type II CRISPR-Cas nuclease Cas9 [4], and further experiments revealed the usefulness of these systems for genome engineering [5, 6]. Cas9 can be specifically targeted to any locus of interest, whereupon it cleaves the DNA, stimulating a variety of DNA damage response mechanisms that can lead to knockouts, gene conversions, and gene corrections [7]. Unlike its predecessors (zinc finger nucleases, TAL effector nucleases), however, whose targeting is based on modular, protein-based recognition domains that must be rearranged for each new target site, the Cas9 enzyme recognizes target DNA sequences by Watson–Crick base pairing between its gRNA and the target.

These developments have been broadly welcomed by the research community, which has embraced the *Streptococcus pyogenes* Cas9 (SpCas9), a 1368 amino acid variant whose only targeting limitation is the requirement of a protospacer adjacent motif (PAM) consisting of NGG nucleotides immediately 3′ to the target site [8]. In the few years since its debut, the SpCas9 has been successfully used in a plethora of model and commercially valuable organisms [5, 6, 9–11] and has been the subject of extensive characterizations and modifications. Cas9 nucleases from other bacterial species, such as *Streptococcus thermophilus*, *Neisseria meningitides*, and *Staphylococcus aureus*, have also been identified, and preliminary characterizations reveal substantial variations in size, PAM sequence requirements, and DNA cleavage efficiencies [12, 13]. Here we report further insights and tool developments related to the *S. aureus* Cas9 (SaCas9), a 1053 amino acid protein that may provide substantial advantages due to its size and efficacy [13].

## Results and discussion

**Fig. 1:**
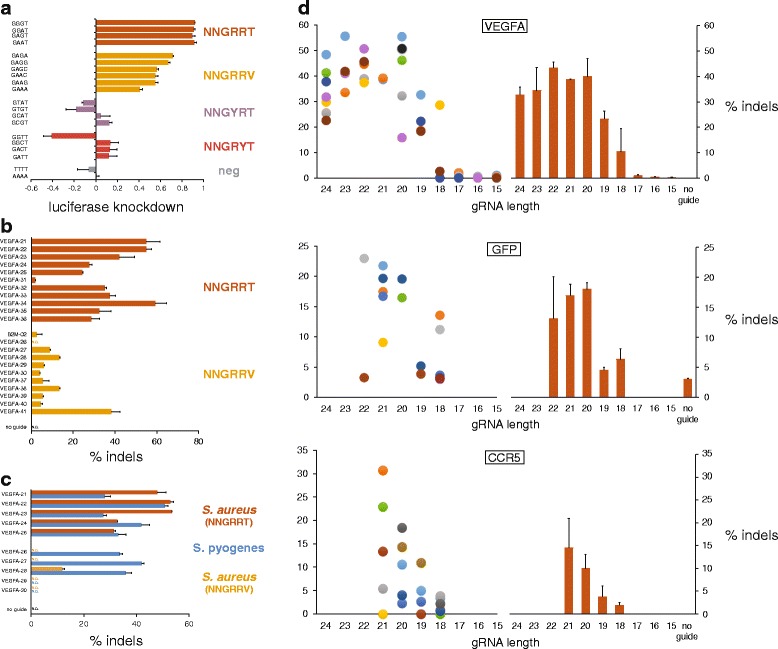
SaCas9 PAM characterization and gRNA spacer length assessment in HEK293, HEK293T, HEK293FT, and HEK293-GFP cells. **a** A plasmid-based, luciferase knockdown assay in which an invariant target sequence with variable PAMs was placed at the 5′ end of luciferase. Data are shown as means ± standard error of the mean (SEM) (*N* = 3). **b** T7E1-measured indel rates resulting from SaCas9 directed to endogenous targets with NNGRRT and NNGRRV PAMs. Data are shown as means ± SEM (*N* = 2). An unpaired t-test for the two groups yields a *P* value of <0.0001. **c** Comparisons of indel rates between SaCas9 and SpCas9 at targets with overlapping (NGGRR(T)) PAMs. Data are shown as means ± SEM (*N* = 2). *N.D.* none detected. **d** Indel rates resulting from SaCas9 directed to endogenous *VEGFA* (*top*) and CCR5 (*bottom*) targets, with gRNAs of varying spacer length. “Sibling” gRNAs target the same precise locus, initiate with a target-matching G, and are marked with *same-colored dots. Orange bars* represent mean cleavage (±SEM (*N* ≥ 3)) for gRNAs of that length. *N.D.* none detected. *Middle*: Knockdown of green fluorescent protein (GFP) in HEK293-GFP cells as measured by percentage of cell population that is GFP-negative 3.5 days post-transfection

To corroborate the reported PAM recognition sequence of NNGRRT [13], we utilized a luciferase reporter assay in which HEK293T cells were transfected with a SaCas9/gRNA dual expression plasmid (pCMVSau) along with one of a series of firefly luciferase indicator plasmids, each containing a different PAM sequence adjacent to the invariant target site [14] (Figure S1 and Table S1 in Additional file 1). Results of this assay, where strong luciferase knockdown indicates robust SaCas9 cleavage of the target plasmid, show highest targeting efficiency at NNGRRT PAMs, and moderate cleavage of targets with NNGRRV PAMs (Fig. 1a). To determine whether this activity profile is maintained at endogenous loci, we designed gRNA constructs directing SaCas9 to target sites in the human *VEGFA* and *B2M* loci with either NNGRRT or NNGRRV PAMs (Fig. 1b). These gRNAs were designed with spacer lengths of 24 nucleotides and a target-matching 5′ G to encourage consistent expression from the U6 promoter. We assayed cleavage of target DNA and the resulting formation of insertions and deletions (indels) that arise via imperfect repair of the DNA through non-homologous end joining (NHEJ) of these double-strand breaks (DSBs). At these endogenous loci, there was a substantial preference for the NNGRRT PAMs, though target sites with NNGRRV PAMs still permitted some nuclease activity.

Because the SaCas9 and SpCas9 PAM sequences are not mutually exclusive, we directly compared the two nucleases at identical target sites with overlapping PAMs consisting of NGGRR(T) (Fig. 1c). SaCas9 gRNAs were 24-mers initiating with a target-matching 5′ G, while SpCas9 gRNAs were 20-mers with an additional 5′ G (21-mers). Results indicate that when the SaCas9 (pAF003) was directed to targets with its preferred NNGRRT PAM, cleavage efficiencies between it and SpCas9 (pAF028) were comparable, with SaCas9 outperforming SpCas9 in some cases. As expected, when SaCas9 was directed to target sites with NNGRRV PAMs, significantly less DNA cleavage was observed compared with SpCas9-induced cleavage.

We next varied the length of the gRNA spacer sequence to determine the optimum for maximal DNA cleavage when in complex with SaCas9. Beginning with a selection of effective 24-mer spacers from Fig. 1b and an additional 23-mer spacer (VEGFA-15) targeting a ~500-bp region of the *VEGFA* gene, we generated all available G-initiating “sibling” gRNAs, which have spacers of different lengths but target the same site. With this strategy, the initial group of 12 24-mer gRNAs expanded to 44 gRNAs with spacer lengths ranging from 24 to 15 bases, all with a target-matching 5′ G. Results from this experiment show that maximum gRNA efficiencies can be achieved with spacer lengths ranging from 24-mers down to 20-mers, that 19-mers and 18-mers can still have some activity, and that 17-mers and shorter are inactive (Fig. 1d, top). To increase the resolution around the 20 base to 17 base window, we designed gRNAs for a second, similar experiment targeting sites at a second locus, *CCR5* (Fig. 1d, bottom). A third such experiment, this time with gRNAs designed to knock out an integrated green fluorescent protein (GFP) from HEK293-GFP cells, was carried out to further increase the total number of sites tested (Fig. 1d, middle). Taken together, these data show the effectiveness of gRNAs across a range of spacer lengths, with sequences of 24 to 20 bases working most efficiently.

**Fig. 2:**
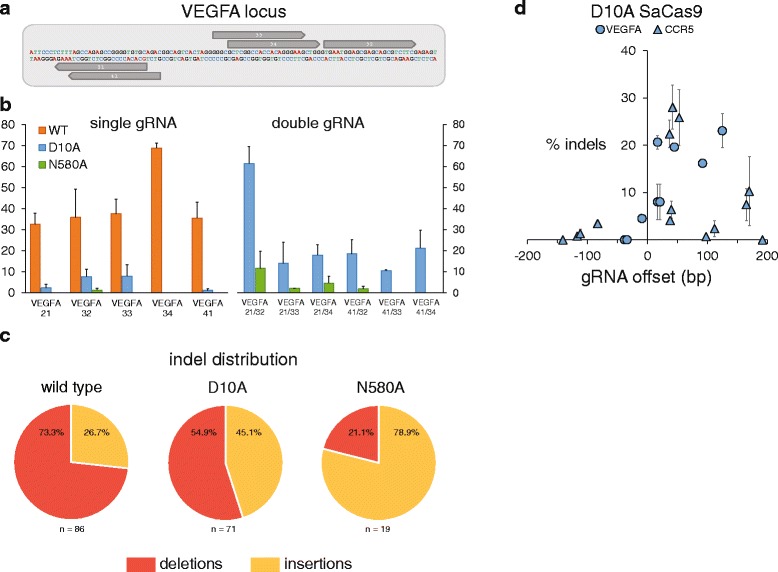
D10A and N580A SaCas9 nickase data. **a** Sequence of the target locus (*VEGFA*) with five gRNAs annotated in *grey*. **b** Wild-type (*WT*) SaCas9, D10A and N580A SaCas9 nickases generating indels with single and dual gRNAs. Percentage indels on the y-axis represents on-target cleavage rates as measured by TOPO sequencing. Data are shown as means ± standard error of the mean (SEM) (*N* = 2). *N.D.* none detected. **c** Indel distribution of insertions and deletions from each SaCas9 type. Wild-type SaCas9 data shown are from transfections with single gRNAs, whereas D10A and N580A data shown are from transfections with dual gRNAs. **d** Indel rates as measured by TOPO sequencing for D10A SaCas9 and pairs of gRNAs with offset distances (between the 5′ ends of the gRNAs) ranging from -150 bp to 200 bp

Next, we constructed two SaCas9 nickases by aligning the amino acid sequence to that of SpCas9 and identifying residues corresponding to the previously described D10A and N863A substitutions [15] (Figure S2 in Additional file 1). We used site-directed mutagenesis to generate D10A and N580A mutants that would similarly disable the RuvC and HNH nuclease domains, respectively. We selected five gRNAs from those shown in Fig. 1b that yielded high levels of indels and whose orientations with respect to each other make them suitable for use as nickase pairs (Fig. 2a), and transfected them individually and as pairs with the wild-type (pAF003), D10A (pAF008), and N580A (pAF009) SaCas9 plasmids. Sequencing of the locus showed that the nickases had minimal activity when transfected with a single gRNA; N580A typically yielded no indels and D10A yielded indel rates between 0 % and ~8 %. Cells that were transfected with a nickase and two gRNAs, on the other hand, yielded indels with a range of efficiencies extending up to ~60 %. Interestingly, the D10A nickase consistently outperformed the N580 nickase (Fig. 2b). Their respective indel distributions were also substantially different, with D10A indels evenly split between insertions and deletions, while N580A indels were predominantly insertions. Wild-type SaCas9 indels were predominantly deletions (Fig. 2c).

To further characterize features of the SaCas9 paired nickases, we selected an array of gRNAs that efficiently induced indels at their targets when expressed with wild-type SaCas9 (Figure S3 and Tables S1 and S2 in Additional file 1) and transfected them with the D10A mutant as pairs with offset distances ranging from approximately -150 bp to 200 bp (Fig. 2d). Pairs of gRNAs with offsets below zero yielded minimal indel rates, while most gRNA pairs with offsets between 0 and 125 bp yielded substantial indel rates. Surprisingly, and in contrast with SpCas9 data previously reported [15], some gRNA pairs with offsets between 125 and 170 bp yielded modest indel rates. These data reflect the general effectiveness of the D10A SaCas9 nickase; however, other properties of the SaCas9 nickases, such as whether they can shift the balance between homology-dependent repair and NHEJ, and/or whether they can reduce off-target DNA cleavage compared with the wild-type nuclease (features reported for other nickases [15, 16]) warrant future attention.

**Fig. 3:**
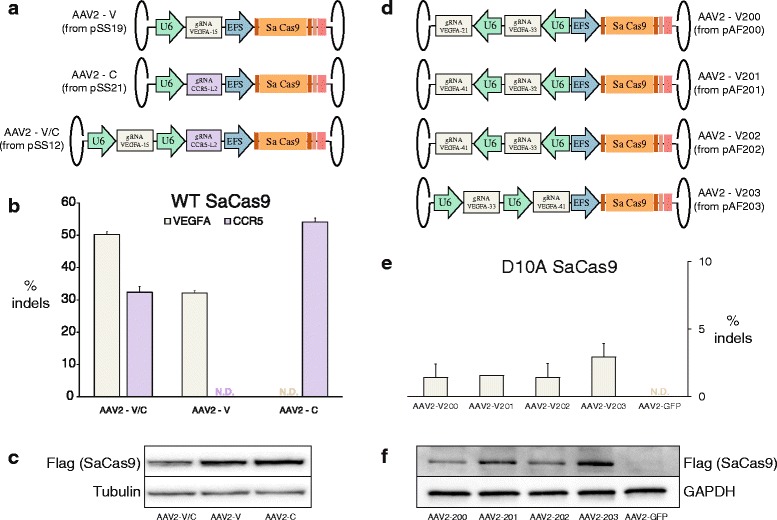
**a**. AAV constructs containing SaCas9 with a U6 promoter driving a VEGFA-15 gRNA, with a U6 promoter driving a CCR5-L2 gRNA, and with both U6-gRNAs. **b** Indel rates at *VEGFA* and *CCR5* loci as measured by T7E1. Data are shown as means ± standard error of the mean (SEM) (*N* ≥ 3). *N.D.* none detected. **c** Western blots for Flag (SaCas9) and Tubulin confirming expression of SaCas9 in transduced cells. **d** AAV constructs containing D10A SaCas9 with different pairs of *VEGFA*-targeting gRNA expression cassettes. **e** Indel rates at *VEGFA* as measured by TOPO cloning and sequencing. Data are shown as means ± SEM (*N* = 2). *N.D.* none detected. **f** Western blots for Flag (SaCas9) and GAPDH confirming expression of D10A SaCas9 in transduced cells

We also made use of the small size of the SaCas9 and packaged it along with two U6-promoter-driven gRNAs in a single adeno-associated virus (AAV). The SaCas9 is encoded by 3159 bp, leaving ~1.8 kb of space for regulatory elements and gRNAs given the ~5.0 kb packaging limit of AAVs [17]. Here, we drove the SaCas9 expression with an EFS promoter and added a mini poly(A) tail to its 3′ end (Fig. 3a). We made seven such vectors: the first with the SaCas9 expression cassette (with its EFS promoter and poly(A) tail) and a U6-promoter-driven gRNA targeting *VEGFA*, the second with the SaCas9 cassette and a U6-promoter-driven gRNA targeting *CCR5*, and the third with the SaCas9 expression cassette and both the aforementioned gRNA cassettes. The other four vectors we constructed contained D10A SaCas9 expression cassettes along with various pairs of *VEGFA*-targeting gRNA cassettes (Fig. 3d). We packaged these AAVs with a serotype 2 capsid, then transduced HEK293 and HEK293FT cells. Western blots confirmed the expression of SaCas9 in all transduced samples (Fig. 3c, f), while T7E1 assays and sequencing showed a range of Cas9 activity at targeted loci (Fig. 3b, e, respectively).

This ability to package two gRNAs and SaCas9 in a single AAV opens the door to additional gene editing approaches using “all-in-one” AAV vectors, such as targeted deletions mediated by wild-type SaCas9 and two gRNAs, and multiplexed knockouts. With the current total vector length of 4859 bp, it is conceivable that a third gRNA could be added to these vectors given further reductions in the size of the regulatory sequences used. To this end, it may be helpful to use human tRNA promoters, which are only ~70 bp in size, and were recently demonstrated to be as effective as the ~250 bp U6 promoter used here in driving gRNA expression [18]. Moreover, it may also be possible to delete some non-essential sequences from the SaCas9 gene itself without reducing nuclease activity or specificity.

**Fig. 4:**
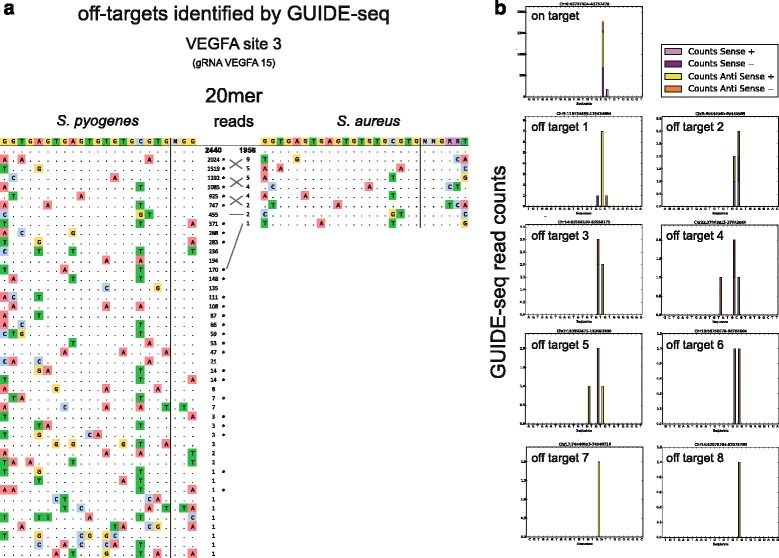
**a** Sequences of off-target sites identified by GUIDE-seq for SpCas9 and SaCas9 when directed by gRNA to VEGFA site 3. The intended target sequence is shown in the top line with the guide sequence on the left and the PAM on the right. On-target reads are shown in *bold*, and mismatches found in off-target sequences are highlighted in *color*. Off-target sites found in both datasets are matched with *grey lines*. Previously identified SpCas9 off-target sites are indicated with *asterisks*. **b** Histograms of mapped reads at off-target loci

Finally, we conducted a GUIDE-seq experiment (Fig. 4a, b) to assay SaCas9 specificity compared with SpCas9 with a gRNA that has a target (*VEGFA* site 3) with known off-target sites [19, 20]. Our experiment, in which each Cas9 was directed by a gRNA whose spacer length is 20 bases, identified a substantial number of the SpCas9 off-target sites found previously, with read counts at some off-target sites nearly as high as those for the on-target site. In contrast, our GUIDE-seq results for SaCas9 show a high number of on-target reads but only single-digit read counts for comparatively fewer off-targets. Interestingly, all eight of the SaCas9 off-target sites identified in this experiment were also identified as off-targets for SpCas9.

Additional experiments will be required to accurately determine the indel rates at these off-target sites; however, the read counts suggest that SaCas9 is, in this case, more specific than SpCas9 when directed by a 20-bp spacer. To further support the use of SaCas9 for genome engineering, it will be important to use such minimally biased techniques to evaluate its specificity at other loci, and with gRNAs of a variety of lengths. Existing BLESS data similarly indicate SaCas9 is more specific than SpCas9 at two other target loci, but this could vary by gRNA and target sequence [13]. These and other characterizations are likely to be of strong interest to the genome engineering community given the array of in vitro, in vivo, and therapeutic applications that may be facilitated by this smaller, high-efficiency Cas9.

## Conclusions

The *S. aureus* Cas9 has a number of properties that make it advantageous for genome editing, including its small size, high efficiency, nickase activity, and apparent specificity. Here we characterized a number of salient features of the wild-type SaCas9 as well as the nickase variants that we engineered.

We found that SaCas9 principally recognizes an NNGRRT PAM, and that it cleaves target DNA at rates comparable to that of SpCas9. Further, we explored the gRNA spacer lengths necessary for SaCas9-mediated target cleavage, finding robust activity when spacer lengths were 24 to 20 nucleotides. Building on these findings, we generated SaCas9 nickases and assayed their functionality with a set of suitable gRNA pairs. The D10A nickase consistently displayed more activity than the N580A nickase, inducing indels with frequencies as high as ~60 %. We explored the efficiency of D10A SaCas9 with gRNA pairs at a range of distances, finding activity with pairs offset from ~0 to ~170 bp.

We also highlighted one of the major advantages of SaCas9, exploiting its small size to package it in an AAV with two gRNAs. These viruses were effective at both simultaneously inducing indels in multiple genes with the wild-type SaCas9 as well as inducing indels at single target loci with the D10A SaCas9.

Finally, we performed specificity experiments using the GUIDE-seq technique. Our results corroborate previously identified SpCas9 off-target sites for a known gRNA, and we moreover found that SaCas9 induces DSBs at a subset of these sites with significantly lower read counts.

Taken together, our characterizations and manipulations of SaCas9 reveal a valuable set of tools for a wide array of CRISPR-based genome engineering applications.

## Materials and methods

### Cell types used

**Table:** 

Fig. 1a (luciferase)	Cell type: HEK293T
Fig. 1b (NNGRR(T/V))	Cell type: HEK293
Fig. 1c (SaCas9 vs SpCas9)	Cell type: HEK293FT
Fig. 1d (top) (gRNA length — *VEGFA*)	Cell type: HEK293
Fig. 1d (mid) (gRNA length — *GFP*)	Cell type: HEK293-GFP
Fig. 1d (bottom) (gRNA length — *CCR5*)	Cell type: HEK293
Fig. 2b–d (nickases)	Cell type: HEK293FT
Fig. 3a–c (AAV transduction)	Cell type: HEK293
Fig. 3d–f (AAV transduction)	Cell type: HEK293FT
Fig. 4a, b (GUIDE-seq)	Cell type: U-2 OS

### Cell culture

HEK293, HEK293FT (Life Technologies, catalog #R700-07), HEK293-GFP (GenTarget, catalog #SC001), and U2-OS (ATCC #HTB-96) cells were maintained in Dulbecco’s modified Eagle medium (DMEM; Life Technologies) supplemented with 10 % fetal bovine serum (FBS), 5 % penicillin/streptomycin, and 2 mM Glutamax. Cells were kept at 37 °C in a 5 % CO_2_ incubator.

### Plasmid and gRNA construction

The pCMVSau plasmid expressing a human codon optimized SaCas9 and a customizable U6-driven gRNA scaffold have been previously described [18]. Cognate luciferase indicator constructs were generated as previously described [14]. Maps of these plasmids and all other SaCas9 plasmids are shown in Figure S1 in Additional file 1.

gRNA used in Fig. 1a was generated by cloning annealed oligos containing the target sequence into pCMVSau. gRNAs used for data shown in Figs. 1b–d and 2d were generated by PCR and transfected as amplicons containing U6 promoter, spacer sequence, and TRACR scaffold. gRNAs used for data shown in Figs. 2b, c and 4a, b were generated by ligating either one or two of these into a pUC19 backbone vector via Gibson Assembly (New England Biolabs).

AAV vectors used in Fig. 3a–c were constructed by Gibson Assembly of one or two gRNA cassettes into SaCas9-containing AAV backbone pSS3. Vectors used in Fig. 3d–f were constructed by subcloning gRNA cassette pairs from vectors pAF089, pAF091, pAF092 into pSS60. Inverted terminal repeats (ITRs) were confirmed by XmaI digest of the vectors.

### Transfections

Cells were seeded at a density of 100,000 cells/well in 24-well plates. After 24 hours, cells were transfected with 250 ng of gRNA plasmid or amplicon and 750 ng of either wild-type Cas9 plasmid, Cas9-D10A nickase plasmid, or Cas9-N580A nickase plasmid. All transfections were performed in duplicate using either Lipofectamine 3000 (Life Technologies) or MirusTransIT-293 reagent (Mirus Bio).

### Luciferase analysis

293T cells were seeded at 1.25 × 10^5^ cells per well in 12-well plates. Cells were transfected using the calcium phosphate method with 1 μg of the SaCas9/gRNA expression vector, 250 ng of a cognate gRNA firefly luciferase indicator plasmid, and 10 ng of a renilla luciferase internal control plasmid. Transfected cells were harvested 72 hours post-tranfection and lysed in Passive Lysis Buffer (Promega) and then assayed for luciferase activity using a Dual Luciferase Assay Kit (Promega).

### GFP analysis

At 3.5 days post-transfection, cells had their media removed and were washed with 500 μl of phosphate-buffered saline (PBS). Next, 200 μl of trypsin was added to the cells and they were incubated at 37 °C with 5 % CO_2_ for 5 min. Trypsinization was halted by adding 500 μl of complete media to each well. Cells were collected from each well and transferred to eppendorf tubes, spun down at 3000 rpm for 7 min, washed with 1 ml fluorescence-activated cell sorting (FACS) buffer (PBS with 3 % FBS) and spun down again, and finally resuspended in 200 μl FACS buffer. Cells were then analyzed with a BD Accuri C6 flow cytometer.

### DNA analysis

DNA was harvested 72 hours post-transfection or post-infection using an Agencourt DNAdvance genomic DNA isolation kit (Beckman) with a 4 hour lysing period, according to the manufacturer’s directions. Genomic DNA was then purified using Agencourt AMPure XP beads (Beckman) as per the manufacturer’s protocol.

For T7E1 assays, locus PCRs were performed to amplify regions of *VEGFA*, *CCR5*, and *B2M*. All reactions were performed with Phusion high-fidelity DNA polymerase (New England Biolabs) with resulting products purified by Agencourt AMPure XP beads (Beckman) according to the manufacturer’s instructions. T7E1 digestion was then performed in NEB Buffer 2 according to manufacturer’s instructions and resulting cleavage products were analyzed on a Qiagen QIAxcel Advanced System (Qiagen).

PCR conditions (Table S3 in Additional file 1).

**Table:** 

Locus: VEGF(1)	Primers: OME6/OME8	Annealing Temp: 67.5 °C
Locus: VEGF(2)	Primers: AF116/AF117	Annealing Temp: 64 °C
Locus: CCR5(1)	Primers: AF205/AF208	Annealing Temp: 64 °C
Locus: CCR5(2)	Primers: AF209/AF211	Annealing Temp: 64 °C
Locus: B2M	Primers: GWED67/68	Annealing Temp: 65 °C

For nickase assays, amplified *VEGFA* locus fragments were cloned into pCR4-TOPO vector using ZeroBlunt TOPO Cloning Kit (Life Technologies). TOPO reaction products were then transformed in One Shot Top10 chemically competent *Escherichia coli* cells. Cells were plated on carbenicillin LB agar plates and incubated overnight at 37 °C. Plasmid DNA was sequenced by Macrogen Corp. and Genewiz, Inc. using an M13 forward primer.

### Viral vector production and titration

HEK293 cells were maintained in DMEM supplemented with 10 % FBS, 100 U/ml penicillin, and 100 U/ml streptomycin on 150-mm petri dishes in 5 % CO_2_ at 37 °C incubation. HEK293 cells were split 1:3 at 18 hours prior to transfection. AAV2 vectors were packaged with the “triple transfection” method using three plasmids: (1) 60 μg of pHelper (Cell Biolabs, Inc., San Diego, CA, USA) expressing E2A, E4, and VA from adenovirus; (2) 50 μg of pRC2 expressing *Rep2* and *Cap2* from AAV2 (Cell Biolabs, Inc.); and (3) 30 μg of pSS/pAF plasmids with ITRs from wild-type AAV2 and CRISPR components. Mirus TransIT-293 reagent (420 μl; Mirus Bio LLC, Madison, WI, USA) was mixed with 14 ml of OptiMEM and incubated at room temperature for 10 min before being added to the mixture of three packaging plasmids. After another 10-min incubation, the transfection mix was evenly distributed to five plates of HEK293 cells. At 70 hours post-transfection, supernatants and HEK293 production cells were collected by pelleting and centrifugation. Cell pellets underwent sonication, CsCl ultracentrifugation, and dialysis with 1× PBS to yield recombinant AAV2 viral particles.

To titrate AAV2 preparations, 10 μl of dialyzed viral vector was incubated in 90 μl of DNaseI solution at 37 °C for 1 hour, followed by serial dilution with ddH2O. Droplets were generated with Bio-Rad QX200 using 70 μl of droplet generation oil and 20 μl of samples including probe, saCas9-1-Probe (5′-6FAM-catcgggattacaagcgtggggtatggg-MGB-NFQ-3′), and primers, OliSS67 (5′-gaactacattctggggctgg-3′) and OliSS68 (5′-acgttggcctccttgaacag-3′). PCR reactions were carried out with 40 μl of droplet mix on a regular thermocycler. Droplets were read with Bio-Rad QX200 system to quantify positive and negative droplets. Viral vector titers were obtained by multiplying ddPCR readouts and dilution factors.

### Vector transduction and western blotting

HEK293 cells were plated at a density of 100,000 cells/well in a 24-well plate and transduced with AAV2 vectors packaging U6-driven gRNA and EFS-driven SaCas9 at a multiplicity of infection (MOI) of 10,000 viral genome (vg)/cell. Growth medium was aspirated off the 24-well plate 72 hours post-transduction and cells were lysed with lysis buffer from the Agencourt DNAdvance kit (Beckman Coulter, Brea, CA, USA) followed by genomic DNA (gDNA) extraction, locus PCR (VEGF and CCR5 loci), and T7E1 assay to quantify genomic modification.

For western blotting, cells were lysed with 1× RIPA buffer with 1× cOmplete ULTRA protease inhibitor cocktail (Roche Diagnostics Corporation, Indianapolis, IN, USA) and 1× PhosSTOP phosphatase inhibitor cocktail (Roche Diagnostics Corporation, Indianapolis, IN, SUA) at 72 hours post-transduction. Cells were lysed at 4 °C for 15 min and lysates were spun down at 13.3 krpm for 15 min at 4 °C. Supernatants were collected and protein concentrations were quantified using Pierce BCA protein assay kit (Life Technologies, Carlsbad, CA, USA). Total protein (41.7 μg) was subjected to 4–12 % NuPAGE Bis-Tris gel electrophoresis at 150 V for 75 min. Gel transfer was performed using High Molecular Weight program on the Trans-Blot Turbo Transfer System (BioRad, Hercules, CA, USA). After blotting with 5 % milk in 1× PBS-T, western blots were incubated separately with corresponding primary antibodies overnight: (1) mouse-anti-Flag (clone m2, F3165, Sigma-Aldrich, St Louis, MO, USA) at 1:1000 dilution in 5 % milk in PBS-T, and (2) mouse-anti-alpha tubulin (clone B7, sc-5286, Santa Cruz Biotechnology, Dallas, TX, USA) at 1:200 dilution in 5 % milk in PBS-T. Blots were washed with PBS-T three times prior to incubation with secondary antibody, goat-anti-mouse IgG-HRP (sc-2005, Santa Cruz Biotechnology, Dallas, TX, USA), at 1:5000 dilution in 5 % milk in TBS-T at room temperature for 1 hour. After four washes with 1× PBS-T, western blots were developed with Western Lightning Plus-ECL (Perkin Elmer, Waltham, MA, USA) and imaged.

### Guide-seq

U-2 OS cells were maintained in DMEM (Life Technologies) supplemented with 10 % FBS, 1 % penicillin/streptomycin. Cells were kept at 37 °C in a 5 % CO_2_ incubator. Cells were nucleofected at a density of 200,000/well with 250 ng of gRNA plasmid (pAF015), 500 ng SaCas9 plasmid (pAF003), and 100 pmol dsODN [19] using SE Cell line nucleofection solution and the DN-100 program on a Lonza 4D- nulceofector (V02.16). The nucleofected cells were seeded in 1 ml media in a 24-well plate and media was changed 12 hours post-nucleofection. Cells were grown for 72 hours post-nucleofection and gDNA was harvested using an Agencourt DNAdvance gDNA extraction kit. dsODN integration at the target site was confirmed by restriction fragment length polymorphism assay with NdeI.

gDNA was quantified with the qubit high sensitivity dsDNA assay kit. Roughly 400 ng of gDNA from SpCas9-treated cells and 180 ng of gDNA from SaCas9-treated cells were sheared acoustically via the Covaris m220 instrument to an average length of 500 bp in a total volume of 130 μl 1× TE. The sheared product was concentrated by AMPure (1× ratio) according to the manufacturer's protocol and eluted in 15 μl of 1× TE. One microliter of the product was run on the Agilent Tapestation system using the D1000 tape to confirm appropriate sizing. The remaining 14 μl of the sheared DNA was end-repaired, A-tailed, and adapter ligated. Adapter-ligated product was cleaned via AMPure (0.9×), eluted in 10 μl 1× TE, and split into sense and anti-sense PCR reactions. Post-PCR products were cleaned via AMPure (1.2×) and eluted in 15 μl of 1× TE. A second round of PCR was then conducted to incorporate the P7 illumina adapter and capture bi-directionality of off-target sites based on dsODN incorporated at each site. The final PCR product was cleaned via AMPure (0.7×) and eluted in 30 μl 1× TE. One microliter of each reaction was analyzed via Agilent Tapestation system using the D1000 screen tape and quantified using the qubit high sensitivity dsDNA assay kit. Finally, each reaction was normalized into one library pool and sequenced on the Illumina Miseq according to the manufacturer's protocols.

We analyzed GUIDE-seq data following the method described in Tsai et al. [19]. Reads were aligned to the UCSC hg19 genome assembly using bowtie2 (PMID:22388286). We selected regions passing the bidirectional filter [19] or with reads originating at the presumptive cutting site (three bases away from the PAM).

### Supporting data

MiSeq sequence data gathered for the GUIDE-seq experiment (Fig. 4) were deposited in the Sequence Read Archive (SRA) at NCBI with BioProject number PRJNA298919. The SpCas9 sense, antisense, and barcode data can be accessed via accession numbers SRX1341497, SRX1341608, and SRX1341607, respectively. The SaCas9 sense, antisense, and barcode data can be accessed via accession numbers SRX1341609, SRX1341611, and SRX1341610, respectively.

## Additional file

Additional file 1:
**Supplementary figures and tables, which include plasmid maps, amino acid sequence alignments, indel data, and gRNA sequences.** (DOCX 2159 kb)

## Abbreviations

AAV: adeno-associated virus
bp: base pair
DMEM: Dulbecco’s modified Eagle medium
DSB: double-strand break
FACS: fluorescence-activated cell sorting
FBS: fetal bovine serum
gDNA: genomic DNA
GFP: green fluorescent protein
gRNA: guide RNA
indel: insertion and deletion
ITR: inverted terminal repeat
NHEJ: non-homologous end joining
PAM: protospacer adjacent motif
PBS: phosphate-buffered saline
PCR: polymerase chain reaction
SaCas9: *Staphylococcus aureus* Cas9
SpCas9: *Streptococcus pyogenes* Cas9
