# Supplementary material for: Characterization of Staphylococcus aureus Cas9: a smaller Cas9 for all-in-one adeno-associated virus delivery and paired nickase applications
Source: Genome Biol. 2015 Nov 24;16:257. doi: 10.1186/s13059-015-0817-8 (PMC4657203; doi:10.1186/s13059-015-0817-8)
Supplement: Additional file 1: — Supplementary figures and tables, which include plasmid maps, amino acid sequence alignments, indel data, and gRNA sequences. (DOCX 2159 kb) [file 13059_2015_817_MOESM1_ESM.docx]

**Additional file 1**

**Figure S1**

| pCMVSau (SaCas9 for luciferase assays) | 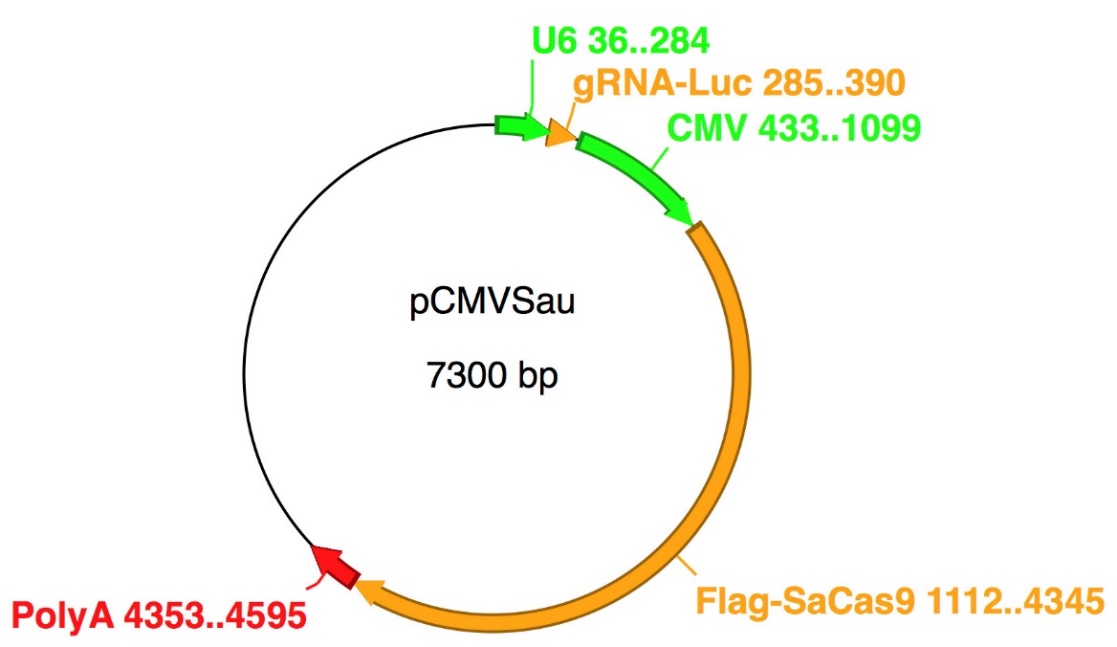 |
| --- | --- |
| pCDNA-Rev-Luc (FLuc target plasmid) | 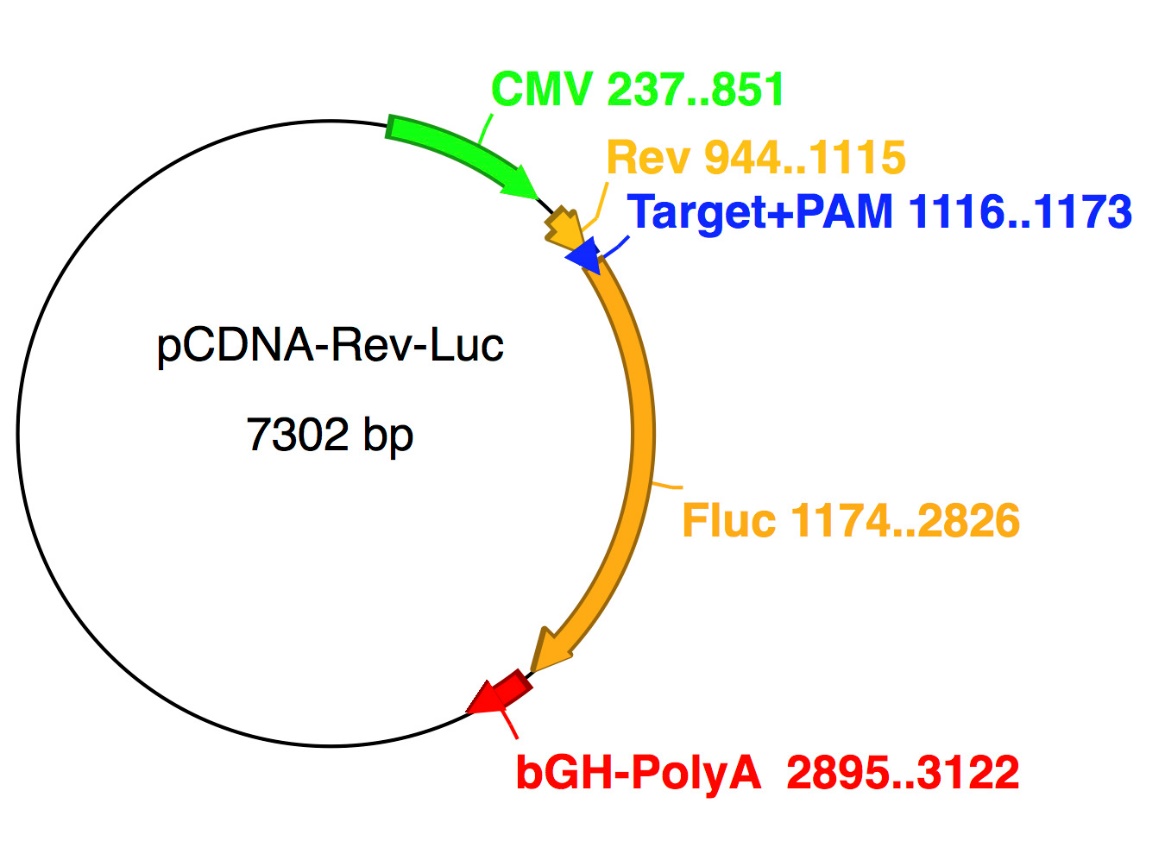 |
| pAF003 (SaCas9) | 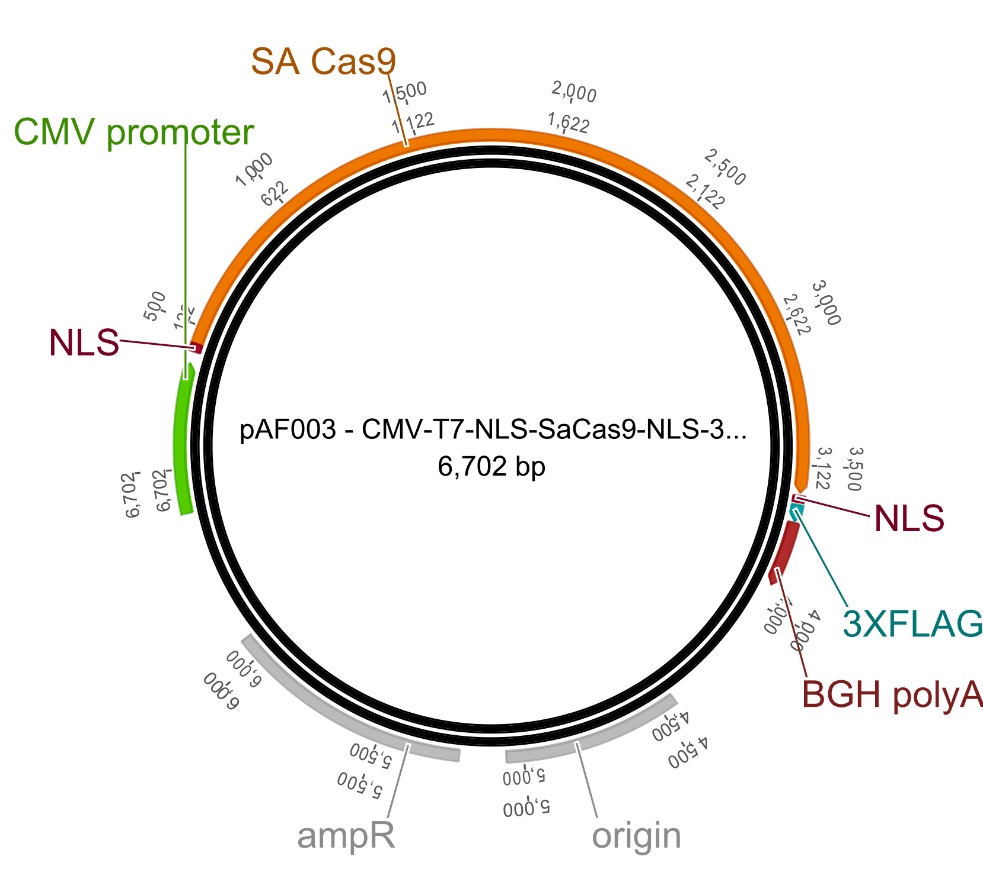 |
| pAF028 (SpCas9) | 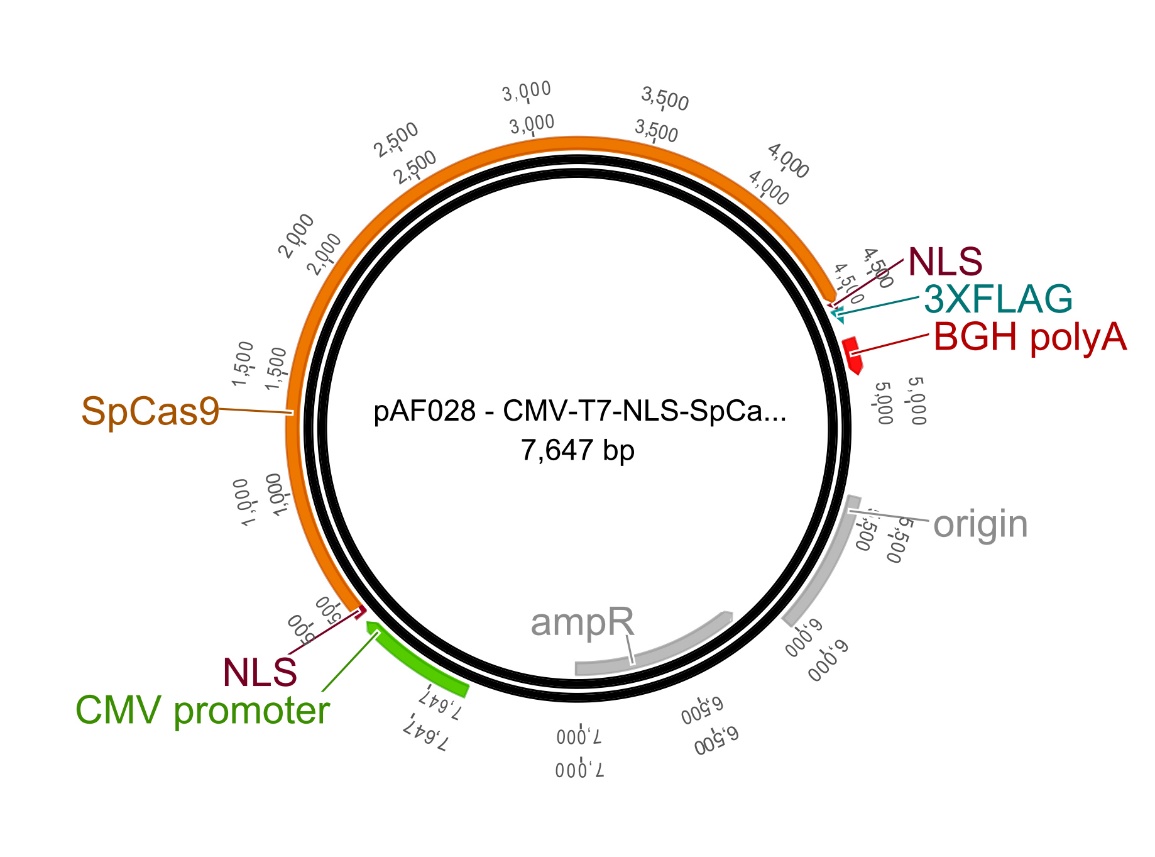 |
| pSS19  (for AAV2 - V) | 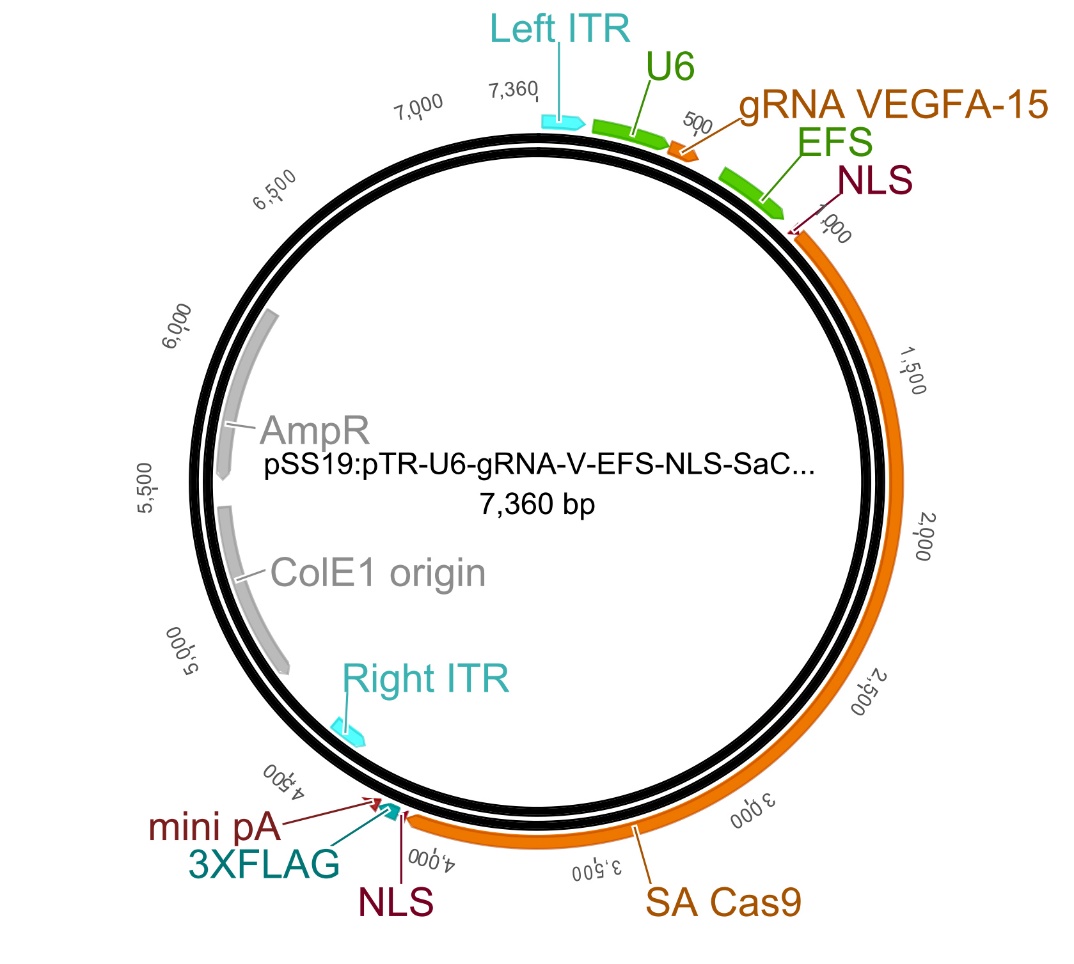 |
| pSS21  (for AAV2 - C) | 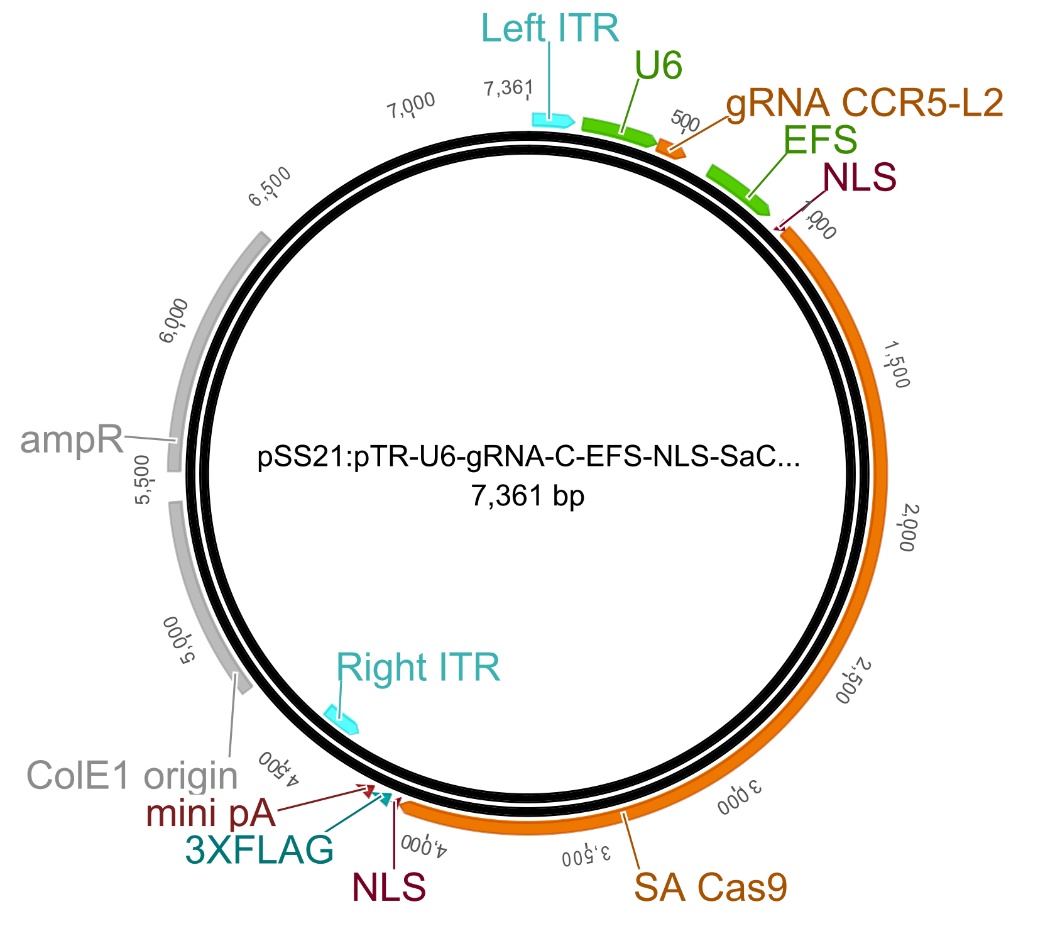 |
| pSS12  (for AAV2 – V/C) | 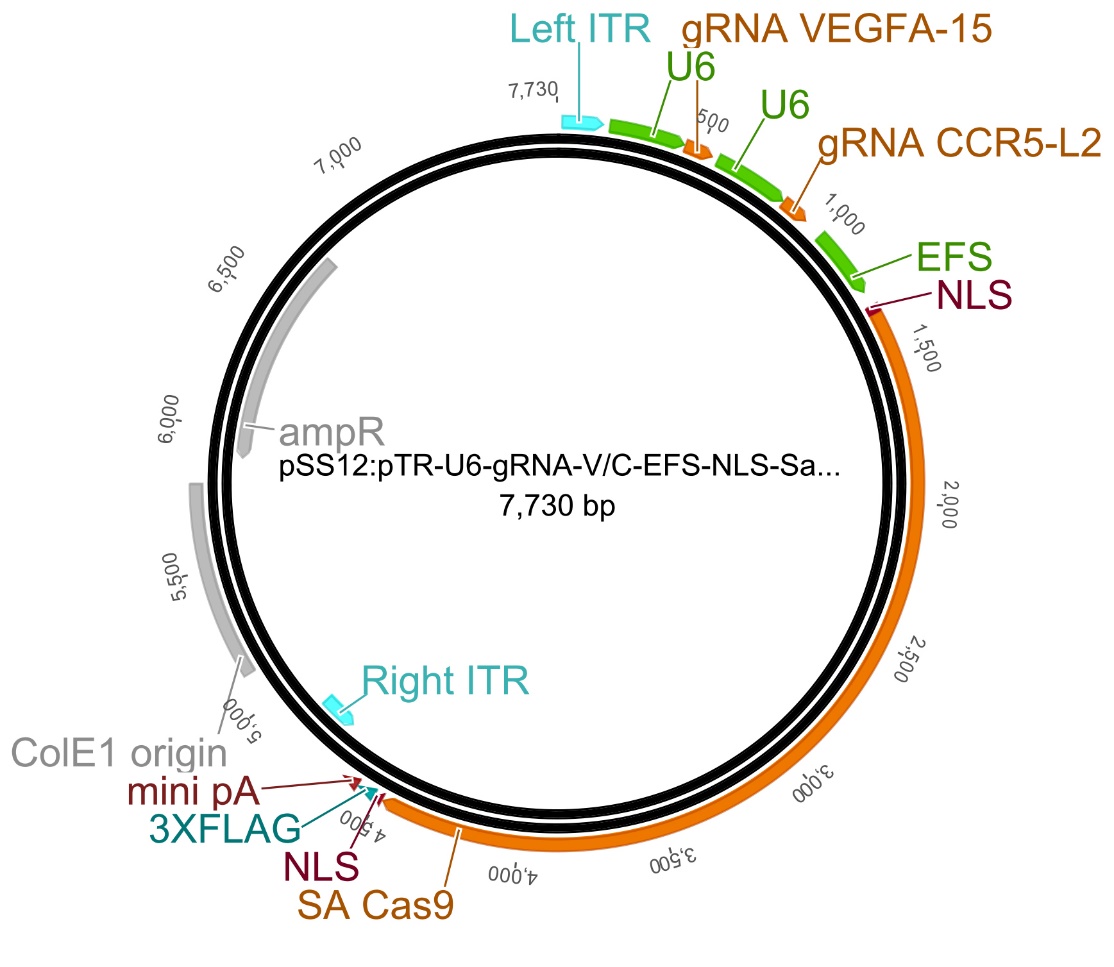 |
| pAF200 (for AAV2-V200) | 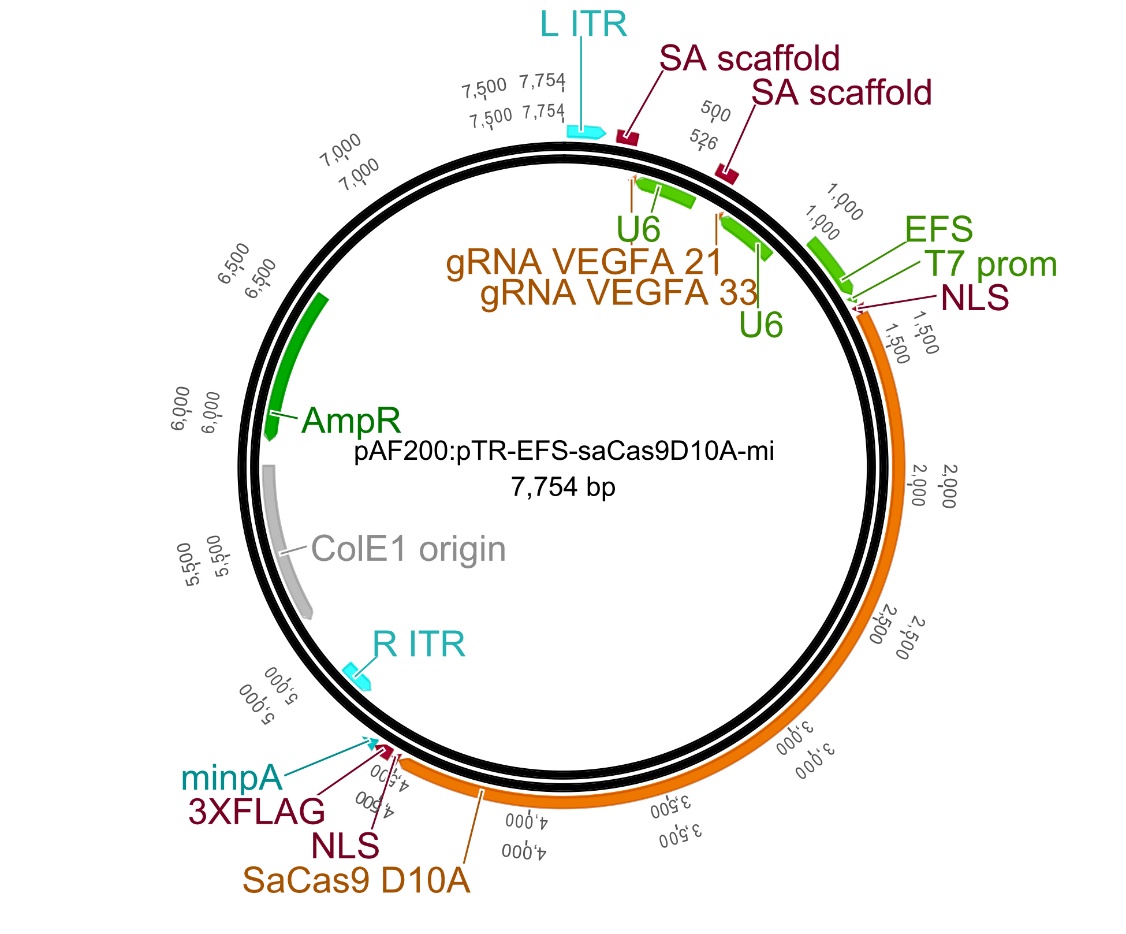 |
| pAF201 (for AAV2-V201) | 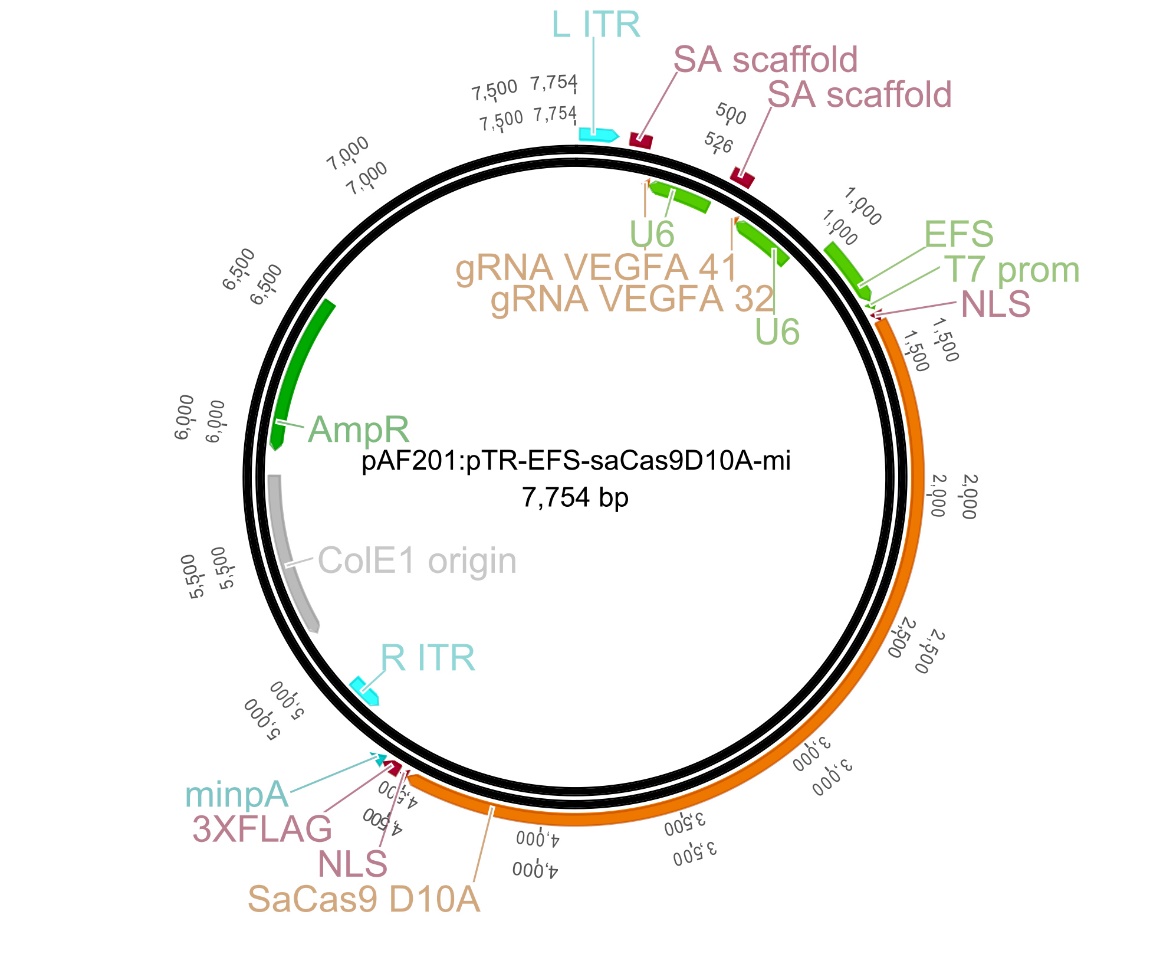 |
| pAF202 (for AAV2-V202) | 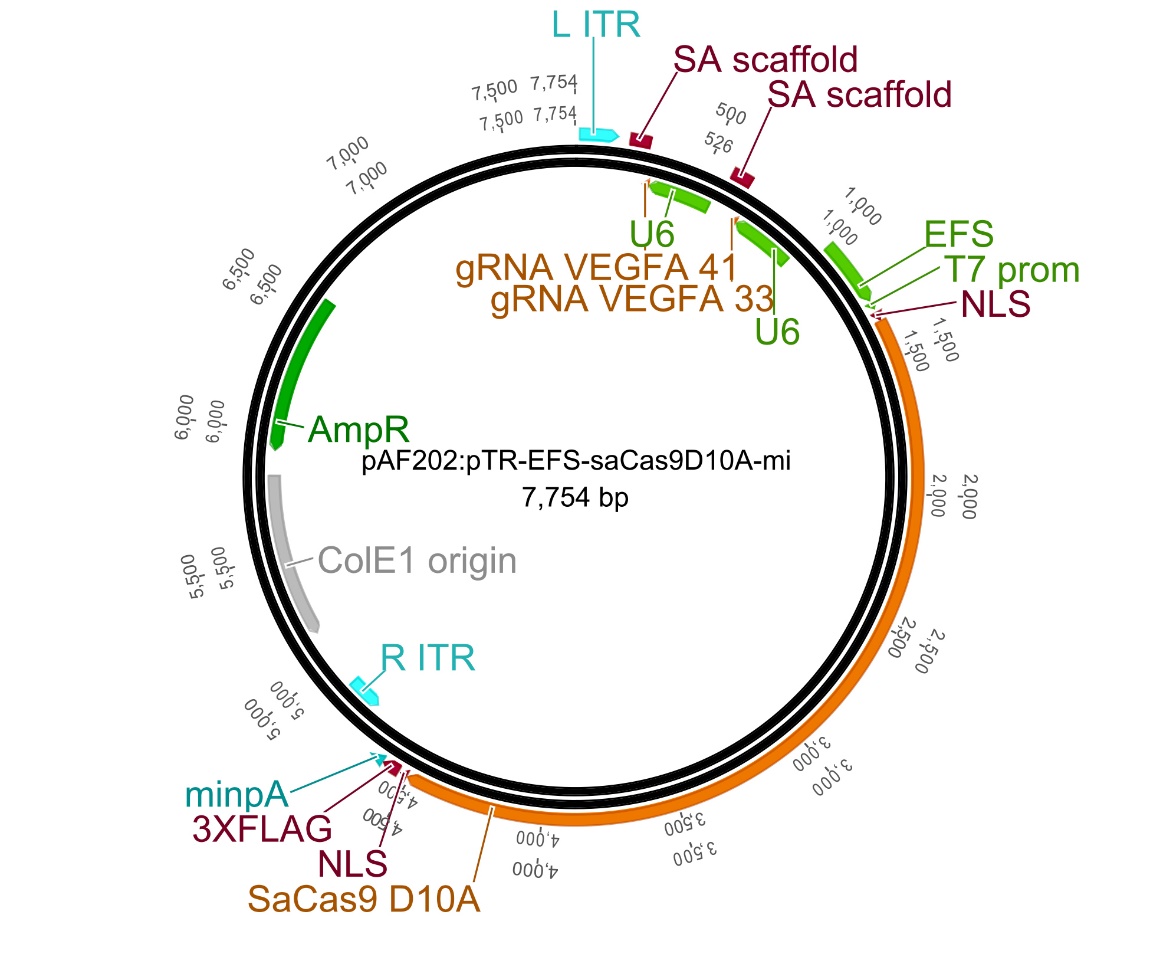 |
| pAF203 (for AAV2-V203) | 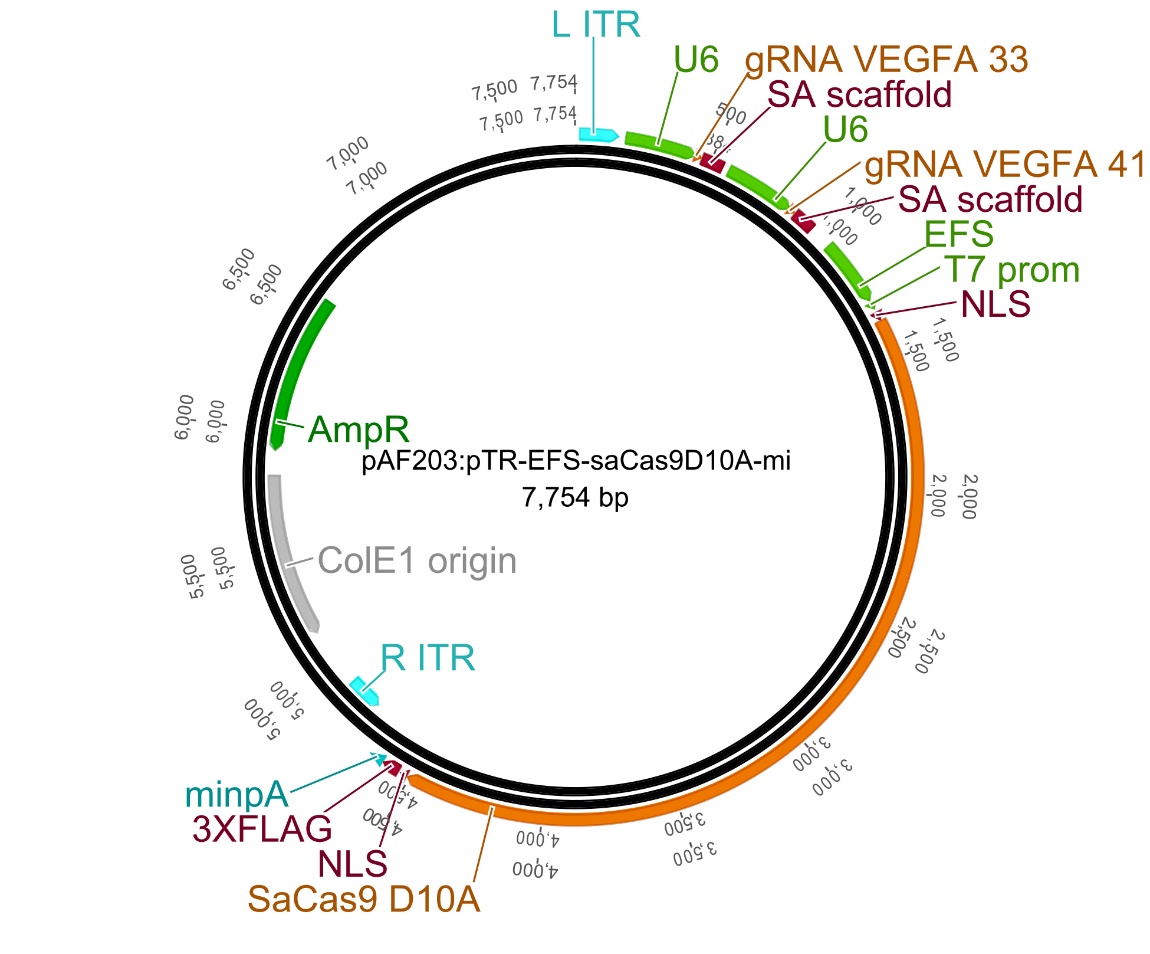 |

**Figure S1**: Maps of some of the Cas9 and AAV plasmids used to generate data shown.

**Figure S2**

**
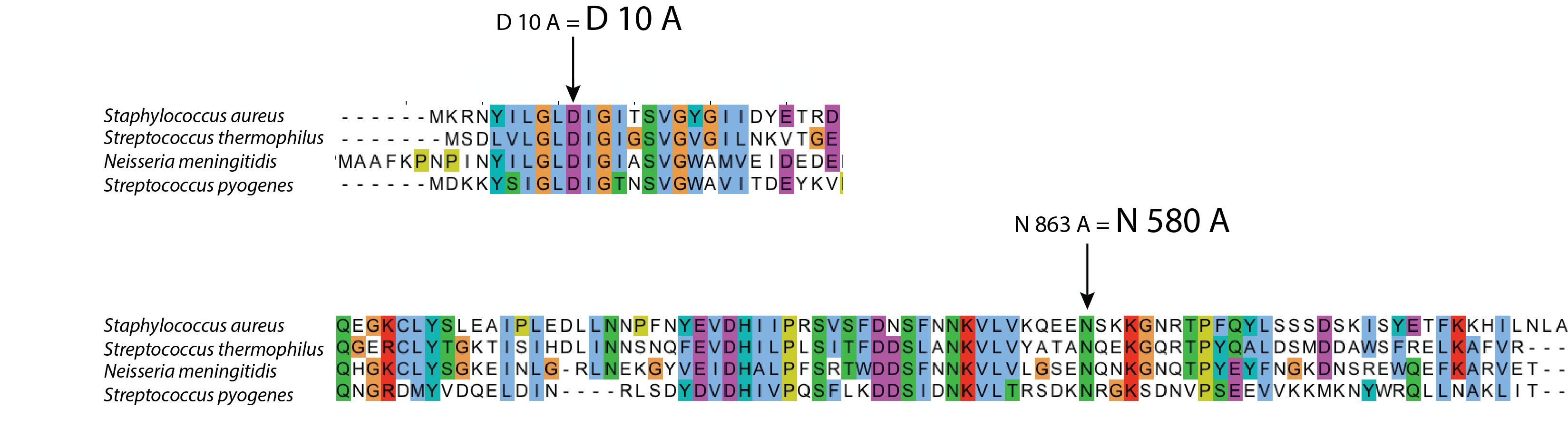
**

**Figure S2**: Partial amino acid alignments of Cas9s from *Staphyloccocus aureus*, *Streptococcus thermophilus*, *Neisseria meningitidis*, and *Streptococcus pyogenes.*

**Figure S3**

**Figure S3**: Indel rates resulting from SaCas9 directed to *CCR5* targets. Data are shown as means ± SEM (*N*=2).

**Table S1**

gRNA sequences

|  |  | Length (bp) |
| --- | --- | --- |
| *S. aureus* TRACR | GTTTTAGTACTCTGGAAACAGAATCTACTAAAACAAGGCAAAATGCCGTGTTTATCTCGTCAACTTGTTGGCGAGATTTTTT | 82 |
| *S. pyogenes* TRACR | GTTTTAGAGCTAGAAATAGCAAGTTAAAATAAGGCTAGTCCGTTATCAACTTGAAAAAGTGGCACCGAGTCGGTGCTTTTTTT | 83 |
|  |  |  |
| **Fig. 1A** |  |  |
| luciferase target | GTGCGCGGCGCATTACCTTTACGC | 24 |
|  |  |  |
| **Fig. 1B** |  |  |
| VEGFA-11 | GGACACACAGATCTATTGGAATCC | 24 |
| VEGFA-21 | GCACACCCCGGCTCTGGCTAAAGA | 24 |
| VEGFA-22 | GCCCATTCCCTCTTTAGCCAGAGC | 24 |
| VEGFA-23 | GGACACACAGATCTATTGGAATCC | 24 |
| VEGFA-24 | GGGGAGAGGGACACACAGATCTAT | 24 |
| VEGFA-25 | GGGCGTTGGAGCGGGGAGAAGGCC | 24 |
| VEGFA-26 | GGGAAGTGTCCAGGGATGCTTCCC | 24 |
| VEGFA-27 | GGAAGTGTCCAGGGATGCTTCCCA | 24 |
| VEGFA-28 | GGGGTGTGCAGACGGCAGTCACTA | 24 |
| VEGFA-29 | GGGCTCCAGATGGCACATTGTCAG | 24 |
| VEGFA-30 | GCGCGTGTCTCTGGACAGAGTTTC | 24 |
| VEGFA-31 | GCCCCGCGGGCGCGTGTCTCTGGA | 24 |
| VEGFA-32 | GTGAATGGAGCGAGCAGCGTCTTC | 24 |
| VEGFA-33 | GGGCGCTCGGCCACCACAGGGAAG | 24 |
| VEGFA-34 | GCTCGGCCACCACAGGGAAGCTGG | 24 |
| VEGFA-35 | GTCCAGGGATGCTTCCCAGGGGAG | 24 |
| VEGFA-36 | GGGAAGGCGGAGAGCCGGACAGGG | 24 |
| VEGFA-37 | GGGACTGGAGTTGCTTCATGTACA | 24 |
| VEGFA-38 | GAGACACGCGCCCGCGGGGCATTG | 24 |
| VEGFA-39 | GACACGCGCCCGCGGGGCATTGGC | 24 |
| VEGFA-40 | GGAGCGAGCAGCGTCTTCGAGAGT | 24 |
| VEGFA-41 | GTCTGCACACCCCGGCTCTGGCTA | 24 |
| VEGFA-42 | GCCTGAGAGCCGTTCCCTCTTTGC | 24 |
| VEGFA-43 | GCCGTTCCCTCTTTGCTAGGAATA | 24 |
| B2M-02 | GGCTGGGCACGCGTTTAATATAAG | 24 |
|  |  |  |
| **Fig. 1C** |  |  |
| SaCas9 VEGFA-21 | GCACACCCCGGCTCTGGCTAAAGA | 24 |
| SpCas9 VEGFA-21 | GACCCCGGCTCTGGCTAAAGA | 21 |
| SaCas9 VEGFA-22 | GCCCATTCCCTCTTTAGCCAGAGC | 24 |
| SpCas9 VEGFA-22 | GATTCCCTCTTTAGCCAGAGC | 21 |
| SaCas9 VEGFA-23 | GGACACACAGATCTATTGGAATCC | 24 |
| SpCas9 VEGFA-23 | GACACAGATCTATTGGAATCC | 21 |
| SaCas9 VEGFA-24 | GGGGAGAGGGACACACAGATCTAT | 24 |
| SpCas9 VEGFA-24 | GAGAGGGACACACAGATCTAT | 21 |
| SaCas9 VEGFA-25 | GGGCGTTGGAGCGGGGAGAAGGCC | 24 |
| SpCas9 VEGFA-25 | GGTTGGAGCGGGGAGAAGGCC | 21 |
| SaCas9 VEGFA-26 | GGGAAGTGTCCAGGGATGCTTCCC | 24 |
| SpCas9 VEGFA-26 | GAGTGTCCAGGGATGCTTCCC | 21 |
| SaCas9 VEGFA-27 | GGAAGTGTCCAGGGATGCTTCCCA | 24 |
| SpCas9 VEGFA-27 | GGTGTCCAGGGATGCTTCCCA | 21 |
| SaCas9 VEGFA-28 | GGGGTGTGCAGACGGCAGTCACTA | 24 |
| SpCas9 VEGFA-28 | GTGTGCAGACGGCAGTCACTA | 21 |
| SaCas9 VEGFA-29 | GGGCTCCAGATGGCACATTGTCAG | 24 |
| SpCas9 VEGFA-29 | GTCCAGATGGCACATTGTCAG | 21 |
| SaCas9 VEGFA-30 | GCGCGTGTCTCTGGACAGAGTTTC | 24 |
| SpCas9 VEGFA-30 | GGTGTCTCTGGACAGAGTTTC | 21 |
|  |  |  |
| **Fig. 1D** |  |  |
| VEGFA-15-23 | GTGGGTGAGTGAGTGTGTGCGTG | 23 |
| VEGFA-15-21 | GGGTGAGTGAGTGTGTGCGTG | 21 |
| VEGFA-15-20 | GGTGAGTGAGTGTGTGCGTG | 20 |
| VEGFA-15-19 | GTGAGTGAGTGTGTGCGTG | 19 |
| VEGFA-15-17 | GAGTGAGTGTGTGCGTG | 17 |
| VEGFA-15-15 | GTGAGTGTGTGCGTG | 15 |
| VEGFA-21-15 | GGCTCTGGCTAAAGA | 15 |
| VEGFA-23-23 | GACACACAGATCTATTGGAATCC | 23 |
| VEGFA-23-15 | GATCTATTGGAATCC | 15 |
| VEGFA-24-23 | GGGAGAGGGACACACAGATCTAT | 23 |
| VEGFA-24-22 | GGAGAGGGACACACAGATCTAT | 22 |
| VEGFA-24-21 | GAGAGGGACACACAGATCTAT | 21 |
| VEGFA-24-19 | GAGGGACACACAGATCTAT | 19 |
| VEGFA-24-17 | GGGACACACAGATCTAT | 17 |
| VEGFA-24-16 | GGACACACAGATCTAT | 16 |
| VEGFA-24-15 | GACACACAGATCTAT | 15 |
| VEGFA-25-23 | GGCGTTGGAGCGGGGAGAAGGCC | 23 |
| VEGFA-25-22 | GCGTTGGAGCGGGGAGAAGGCC | 22 |
| VEGFA-25-20 | GTTGGAGCGGGGAGAAGGCC | 20 |
| VEGFA-25-17 | GGAGCGGGGAGAAGGCC | 17 |
| VEGFA-25-16 | GAGCGGGGAGAAGGCC | 16 |
| VEGFA-32-22 | GAATGGAGCGAGCAGCGTCTTC | 22 |
| VEGFA-32-18 | GGAGCGAGCAGCGTCTTC | 18 |
| VEGFA-32-17 | GAGCGAGCAGCGTCTTC | 17 |
| VEGFA-32-15 | GCGAGCAGCGTCTTC | 15 |
| VEGFA-33-23 | GGCGCTCGGCCACCACAGGGAAG | 23 |
| VEGFA-33-22 | GCGCTCGGCCACCACAGGGAAG | 22 |
| VEGFA-33-20 | GCTCGGCCACCACAGGGAAG | 20 |
| VEGFA-33-16 | GGCCACCACAGGGAAG | 16 |
| VEGFA-33-15 | GCCACCACAGGGAAG | 15 |
| VEGFA-34-20 | GGCCACCACAGGGAAGCTGG | 20 |
| VEGFA-34-19 | GCCACCACAGGGAAGCTGG | 19 |
| VEGFA-35-19 | GGGATGCTTCCCAGGGGAG | 19 |
| VEGFA-35-18 | GGATGCTTCCCAGGGGAG | 18 |
| VEGFA-35-17 | GATGCTTCCCAGGGGAG | 17 |
| VEGFA-36-23 | GGAAGGCGGAGAGCCGGACAGGG | 23 |
| VEGFA-36-22 | GAAGGCGGAGAGCCGGACAGGG | 22 |
| VEGFA-36-19 | GGCGGAGAGCCGGACAGGG | 19 |
| VEGFA-36-18 | GCGGAGAGCCGGACAGGG | 18 |
| VEGFA-36-16 | GGAGAGCCGGACAGGG | 16 |
| VEGFA-36-15 | GAGAGCCGGACAGGG | 15 |
| VEGFA-41-20 | GCACACCCCGGCTCTGGCTA | 20 |
| CCR5-L1-20 | GGGAGGGTAGCATGGTAGTT | 20 |
| CCR5-L1-19 | GGAGGGTAGCATGGTAGTT | 19 |
| CCR5-L1-18 | GAGGGTAGCATGGTAGTT | 18 |
| CCR5-L2-21 | GATGTAGTCAGAGTGAAATGG | 21 |
| CCR5-L2-18 | GTAGTCAGAGTGAAATGG | 18 |
| CCR5-L3-21 | GTTGTTGTTGTTGTTGTGAGA | 21 |
| CCR5-L3-18 | GTTGTTGTTGTTGTGAGA | 18 |
| CCR5-L4-21 | GTGCAGGGAGTTTGAGACTCA | 21 |
| CCR5-L4-19 | GCAGGGAGTTTGAGACTCA | 19 |
| CCR5-L5-20 | GGTTATAAGACTAAACTACC | 20 |
| CCR5-L5-19 | GTTATAAGACTAAACTACC | 19 |
| CCR5-L6-21 | GTTGCCCTAAGGATTAAATGA | 21 |
| CCR5-L6-18 | GCCCTAAGGATTAAATGA | 18 |
| CCR5-L7-20 | GTGAGAGGATTGCTTGAGCC | 20 |
| CCR5-L7-18 | GAGAGGATTGCTTGAGCC | 18 |
| CCR5-L8-21 | GCGTCAATAAAAATGTTAAGA | 21 |
| CCR5-L8-19 | GTCAATAAAAATGTTAAGA | 19 |
| CCR5-L9-20 | GTGCTCTATAGCACCATGGA | 20 |
| CCR5-L9-18 | GCTCTATAGCACCATGGA | 18 |
| CR5-L10-20 | GGAGAGCTTGGCTCTGTTGG | 20 |
| CR5-L10-19 | GAGAGCTTGGCTCTGTTGG | 19 |
| GFP1-21 | GTTGTACTCCAGCTTGTGCCC | 21 |
| GFP1-18 | GTACTCCAGCTTGTGCCC | 18 |
| GFP2-21 | GCCGGTGGTGCAGATGAACTT | 21 |
| GFP2-18 | GGTGGTGCAGATGAACTT | 18 |
| GFP3-22 | GCAAGGGCGAGGAGCTGTTCAC | 22 |
| GFP3-18 | GGGCGAGGAGCTGTTCAC | 18 |
| GFP4-21 | GCCGTAGGTCAAGGTGGTCAC | 21 |
| GFP5-21 | GCTGCCGTCCTCGATGTTGTG | 21 |
| GFP5-18 | GCCGTCCTCGATGTTGTG | 18 |
| GFP6-20 | GTCGTGCTGCTTCATGTGGT | 20 |
| GFP7-21 | GGGGTCTTTGCTCAGGGCGGA | 21 |
| GFP7-20 | GGGTCTTTGCTCAGGGCGGA | 20 |
| GFP7-19 | GGTCTTTGCTCAGGGCGGA | 19 |
| GFP7-18 | GTCTTTGCTCAGGGCGGA | 18 |
| GFP8-22 | GCAGCACGGGGCCGTCGCCGAT | 22 |
| GFP8-19 | GCACGGGGCCGTCGCCGAT | 19 |
| GFP9-18 | GTCGCCGTCCAGCTCGAC | 18 |
|  |  |  |
|  |  |  |
| **Figs. 2A, 2B, 2C** |  |  |
| VEGFA 21 | GCACACCCCGGCTCTGGCTAAAGA | 24 |
| VEGFA 32 | GTGAATGGAGCGAGCAGCGTCTTC | 24 |
| VEGFA 33 | GGGCGCTCGGCCACCACAGGGAAG | 24 |
| VEGFA 34 | GCTCGGCCACCACAGGGAAGCTGG | 24 |
| VEGFA 41 | GTCTGCACACCCCGGCTCTGGCTA | 24 |
|  |  |  |
|  |  |  |
| **Fig 2D** |  |  |
| VEGFA 15 | TGTGGGTGAGTGAGTGTGTGCGTG | 24 |
| VEGFA 21 | GCACACCCCGGCTCTGGCTAAAGA | 24 |
| VEGFA 22 | GCCCATTCCCTCTTTAGCCAGAGC | 24 |
| VEGFA 23 | GGACACACAGATCTATTGGAATCC | 24 |
| VEGFA 25 | GGGCGTTGGAGCGGGGAGAAGGCC | 24 |
| VEGFA 28 | GGGGTGTGCAGACGGCAGTCACTA | 24 |
| VEGFA 31 | GCCCCGCGGGCGCGTGTCTCTGGA | 24 |
| VEGFA 32 | GTGAATGGAGCGAGCAGCGTCTTC | 24 |
| VEGFA 33 | GGGCGCTCGGCCACCACAGGGAAG | 24 |
| VEGFA 34 | GCTCGGCCACCACAGGGAAGCTGG | 24 |
| VEGFA 36 | GGGAAGGCGGAGAGCCGGACAGGG | 24 |
| VEGFA 41 | GTCTGCACACCCCGGCTCTGGCTA | 24 |
| CCR5n-L07 | GCCACTTGGAGGGGTGAGGTGA | 22 |
| CCR5n-L08 | GCCTGTAGTCCCCAGCCACTTGG | 23 |
| CCR5n-L11 | GCGTCAATAAAAATGTTAAGA | 21 |
| CCR5n-L12 | GCCCTAAGGATTAAATGAATGA | 22 |
| CCR5n-L13 | GAGGGAGGGTAGCATGGTAGTT | 22 |
| CCR5n-L14 | GCTGGTTATAAGACTAAACTACC | 23 |
| CCR5n-R12 | GCCTCAGCCTCCTAGAATGC | 20 |
| CCR5n-R14 | GACAAGGACCTTGAAGCACAGA | 22 |
| CCR5n-R15 | GGACCTTGAAGCACAGAGAGG | 21 |
| CCR5n-R16 | GATGTTTGGTGACATGATGTAGT | 23 |
| CCR5n-R17 | GATGTAGTCAGAGTGAAATGG | 21 |
| CCR5n-R18 | GTGAAGTTTGTGTTTGTAGTTT | 22 |
| CCR5n-R19 | GTAGTTTCTGAGTTGCCACAATT | 23 |
| CCR5n-R20 | GAATTTTCTCTTATTAAACCCT | 22 |
|  |  |  |
|  |  |  |
| **Fig 3A-C** |  |  |
|  |  |  |
| CCR5-L2-21 | GATGTAGTCAGAGTGAAATGG | 21 |
| VEGF-15-20 | GGTGAGTGAGTGTGTGCGTG | 20 |
|  |  |  |
| **Fig 3D-F** |  |  |
| VEGFA 21 | GCACACCCCGGCTCTGGCTAAAGA | 24 |
| VEGFA 32 | GTGAATGGAGCGAGCAGCGTCTTC | 24 |
| VEGFA 33 | GGGCGCTCGGCCACCACAGGGAAG | 24 |
| VEGFA 41 | GTCTGCACACCCCGGCTCTGGCTA | 24 |
|  |  |  |
| **Fig 4A-B** |  |  |
| VEGFA-15-20 | GGTGAGTGAGTGTGTGCGTG | 20 |
|  |  |  |

**Table S1**: gRNA TRACR and target (spacer) sequences.

**Table S2**

sgRNAs pairs used for nickase data shown in Figure 2D

| left gRNA | left gRNA  WT SaCas9  indel % | right gRNA | right gRNA  WT SaCas9  indel % | offset length (bp) | left gRNA and right gRNA  D10A SaCas9  indel % |
| --- | --- | --- | --- | --- | --- |
| VEGFA 21 | 55.01 ± 6.53 | VEGFA 22 | 55.05 ± 2.33 | -34 | 0.00 ± 0 |
| VEGFA 21 | 55.01 ± 6.53 | VEGFA 25 | 24.37 ± 0.47 | 125 | 23.04 ± 3.60 |
| VEGFA 21 | 55.01 ± 6.53 | VEGFA 28 | 13.38 ± 0.41 | -9 | 4.50 ± 0.75 |
| VEGFA 21 | 55.01 ± 6.53 | VEGFA 32 | 35.19 ± 0.81 | 45 | 19.62 ± 0.27 |
| VEGFA 21 | 55.01 ± 6.53 | VEGFA 33 | 37.42 ± 2.89 | 17 | 8.02 ± 3.76 |
| VEGFA 21 | 55.01 ± 6.53 | VEGFA 34 | 59.35 ± 5.24 | 21 | 16.54 ± 3.54 |
| VEGFA 41 | 38.12 ± 4.31 | VEGFA 15 | 70.29 ± 4.04 | 92 | 16.18 ± 0.91 |
| VEGFA 41 | 38.12 ± 4.31 | VEGFA 22 | 55.05 ± 2.33 | -38 | 0.00 ± 0 |
| VEGFA 41 | 38.12 ± 4.31 | VEGFA 34 | 59.35 ± 5.24 | 17 | 20.64 ± 1.44 |
| CCR5n-L07 | 20.85 ± 0.22 | CCR5n-R12 | 11.78 ± 1.83 | 112 | 2.34 ± 1.66 |
| CCR5n-L08 | 18.62 ± 1.71 | CCR5n-R12 | 11.78 ± 1.83 | 98 | 0.66 ± 0.47 |
| CCR5n-L11 | 38.18 ± 1.90 | CCR5n-R12 | 11.78 ± 1.83 | -83 | 3.45 ± 0.39 |
| CCR5n-L11 | 38.18 ± 1.90 | CCR5n-R14 | 23.18 ± 3.38 | 165 | 7.43 ± 3.34 |
| CCR5n-L11 | 38.18 ± 1.90 | CCR5n-R15 | 30.83 ± 1.54 | 170 | 10.27 ± 7.26 |
| CCR5n-L12 | 46.37 ± 1.00 | CCR5n-R14 | 23.18 ± 3.38 | 37 | 22.40 ± 2.88 |
| CCR5n-L12 | 46.37 ± 1.00 | CCR5n-R15 | 30.83 ± 1.54 | 42 | 28.02 ± 4.66 |
| CCR5n-L12 | 46.37 ± 1.00 | CCR5n-R16 | 24.76 ± 3.79 | 192 | 0.00 ± 0 |
| CCR5n-L13 | 14.65 ± 0.75 | CCR5n-R14 | 23.18 ± 3.38 | -117 | 0.78 ± 0.55 |
| CCR5n-L13 | 14.65 ± 0.75 | CCR5n-R15 | 30.83 ± 1.54 | -112 | 1.30 ± 0.92 |
| CCR5n-L13 | 14.65 ± 0.75 | CCR5n-R16 | 24.76 ± 3.79 | 38 | 4.09 ± 0.18 |
| CCR5n-L13 | 14.65 ± 0.75 | CCR5n-R17 | 27.76 ± 0.67 | 53 | 25.84 ± 5.97 |
| CCR5n-L14 | 21.17 ± 0.68 | CCR5n-R18 | 11.95 ± 0.14 | -141 | 0.00 ± 0 |
| CCR5n-L14 | 21.17 ± 0.68 | CCR5n-R20 | 32.41 ± 0.30 | 40 | 6.39 ± 1.80 |

**Table S2**: sgRNA pairs and associated data used for Figure 2D. Data are shown as means ± SEM (*N*=2). *VEGFA* gRNAs, *CCR5* gRNAs, and nickase pairs were tested in three separate experiments.

**Table S3**

Locus primers

| OME6 (VEGF) | CCAGATGAGGGCTCCAGATGG |
| --- | --- |
| OME8 (VEGF) | AAGTGAGGTTACGTGCGGACAG |
| AF116 (VEGF) | GGTTTTGCCAGACTCCACAGTGCATACG |
| AF117 (VEGF) | GCTCTGCGGACGCTCAGTGAAGC |
| GWED 67 (B2M) | GAAGTTCTCCTTCTGCTAGGTAGC |
| GWED 68 (B2M) | GCTTCCCCGAGATCCAGC |
| AF205 (CCR5) | CCTAGAATGTATTTAGTTGCCCTCCATGAATGC |
| AF208 (CCR5) | GATTGGCATCCAGTATGTGCCCTCGAGG |
| AF209 (CCR5) | GACGCTAGAGTTAGCCCACAAAGAACATGC |
| AF211 (CCR5) | CCTCTAATATATCAGTTTCATGGCACAG |

**Table S3**: Primers used for locus PCRs in T7E1 and TOPO analyses.
